# Anti-tumor Effect of *Rhaponticum uniflorum* Ethyl Acetate Extract by Regulation of Peroxiredoxin1 and Epithelial-to-Mesenchymal Transition in Oral Cancer

**DOI:** 10.3389/fphar.2017.00870

**Published:** 2017-11-23

**Authors:** Hui Chen, Chunxiao Wang, Moci Qi, Lihua Ge, Zhenchuan Tian, Jinhua Li, Min Zhang, Min Wang, Linfang Huang, Xiaofei Tang

**Affiliations:** ^1^Division of Oral Pathology, Beijing Institute of Dental Research, Beijing Stomatological Hospital, School of Stomatology, Capital Medical University, Beijing, China; ^2^Institute of Medicinal Plant Development, Chinese Academy of Medical Sciences, Peking Union Medical College, Beijing, China

**Keywords:** *Rhaponticum uniflorum*, oral squamous cell carcinoma, peroxiredoxin1, epithelial-to-mesenchymal transition, traditional Chinese medicine

## Abstract

**Objective:** To explore whether *Rhaponticum uniflorum* (*R. uniflorum*) had anti-tumor effects in oral cancer and investigate the molecular mechanisms involved in these anti-tumor effects.

**Methods:** Chemical compositions of *R. uniflorum* ethyl acetate (RUEA) extracts were detected by ultra-performance liquid chromatography-Q/time-of-flight mass spectrometry (UPLC-Q/TOF-MS), followed by pharmacology-based network prediction analysis. The effects of RUEA extracts on proliferation, apoptosis, migration, and invasion ability of human oral squamous cell carcinoma (OSCC) cell line SCC15 were evaluated by CCK8 assay, Annexin V- fluorescein isothiocyanate/propidium iodide staining, wound healing assay, and Matrigel invasion assay, respectively. The mRNA and protein expression of peroxiredoxin1 (Prx1), the epithelial-to-mesenchymal transition (EMT) marker E-cadherin, vimentin, and Snail were determined by quantitative real-time reverse transcription polymerase chain reaction and western blotting. A mouse xenograft model of SCC15 cells was established to further evaluate the effect of RUEA extracts *in vivo*. Immunohistochemical assessment of Ki67 and terminal deoxynucleotidyl transferase dUTP nick end labeling staining of apoptotic cells were performed on the tumor tissues to assess the effects of RUEA extracts on proliferation and apoptosis.

**Results:** Fourteen compounds were identified from RUEA extracts by UPLC-Q/TOF-MS. The pharmacology-based network prediction analysis showed that Prx1 could be a potential binder of RUEA extracts. In SCC15 cells, RUEA extracts inhibited cell viability, induced apoptosis, and suppressed cell invasion and migration in a concentration-dependent manner. After treatment with RUEA extracts, the mRNA and protein expression of E-cadherin increased, whereas those of Prx1, vimentin, and Snail decreased. RUEA extracts also affected the EMT program and suppressed cell invasion and migration in Prx1 knockdown SCC15 cells. In an OSCC mouse xenograft model, RUEA extracts (25 and 250 mg/kg) significantly inhibited the growth of tumors. Compared with the control group, Ki67 expression was reduced and apoptosis rates were elevated in the transplanted tumors treated with RUEA extracts. RUEA extracts increased the expression of E-cadherin and decreased the expression of Prx1, vimentin, and Snail *in vivo*.

**Conclusion:** RUEA extracts inhibited tumor growth and invasion by reducing Prx1 expression and suppressing the EMT process in OSCC. RUEA extracts may be a potential candidate for OSCC treatment.

## Introduction

Oral cancer is one of the most common cancers, with more than 560,000 new cases and 300,000 deaths reported worldwide annually (Siegel et al., 2014). The 5-year survival rate for head and neck cancer remains consistent at around 50%. The major causes of poor prognostic comprise of cervical lymph nodes and distant metastasis (Warnakulasuriya, 2009; Walk and Weed, 2011). Oral squamous cell carcinoma (OSCC) accounts for almost 95% of all head and neck cancers and can develop from oral precancerous lesions, such as leukoplakia and lichen planus (Warnakulasuriya, 2010). Accumulating evidence has suggested that natural plants used in traditional Chinese medicine (TCM) have outstanding advantages such as efficiency, hypotoxicity, sufficiency, and beneficial effects on the treatment of cancers in clinical practice in China and other countries (Shu et al., 2005). Compared with isolated compounds from natural plants, research suggests that complex containing many active phytochemical components in TCM have unique advantages. The different phytochemicals may simultaneously target multiple molecules/pathways and thus potentially achieve better effects (Chow and Huang, 2010). However, the lack of standardization and insufficient information regarding the molecular mechanisms of herbal products remain primary obstacles preventing the global use of TCMs.

*Rhaponticum uniflorum* (L.) DC. (*R. uniflorum*), a species belonging to the Compositae family, is frequently used for reducing fever, detoxifying, and treating malignant ulcers. This is recorded in Shennong Bencaojing, a Chinese book with 1000s of years of history, fully describings the medical effects of plants as a foundation of TCM. *R. uniflorum* composes of several classes of compounds including phytoecdysones, steroids, terpenoids, thiophenes, and flavones (Zhu et al., 1991). According to the Chinese Pharmacopeia, the root of *R. uniflorum* has antioxidant activity and anti-aging effects (National Ceremonial Committee, 2005). Some studies have shown that *R. uniflorum* exhibits various pharmacological properties including anti-inflammatory, anti-oxidative, immunomodulating, and anti-tumor effects. Jin et al. (2011) found that *R. uniflorum* water extracts (RUWE) could inhibit the growth of transplanted Hepatoma-22 (H22) tumors via improving immune and antioxidative functions. However, the effects and the anti-tumor mechanisms of *R. uniflorum* on OSCC are still poorly understood.

Peroxiredoxin1 (Prx1), as a key member of the peroxiredoxins (Prx) family, plays an important role in scavenging reactive oxygen species (ROS) and is overexpressed in various tumors, including oral cancer (Kim et al., 2008; Cha et al., 2009). Our previous studies showed that Prx1 can promote cell proliferation, invasion, and the epithelial-to-mesenchymal transition (EMT) through its peroxidase activity in OSCC (Zhang et al., 2014). The EMT process triggers tumor invasion and migration by regulating epithelial reprogramming which results in loss of cell–cell adhesion accompanied by an increase in cell mobility. Along with morphological alterations, epithelial cell markers are down-regulated, whereas mesenchymal cytoskeletal proteins and transcription factors are up-regulated, resulting in promoting tumor metastasis (Wu and Zhou, 2009).

Finding efficient methods to investigate the interactions between the chemical components of natural plants and single biological molecules in mechanistic studies of different diseases remains a challenging issue in TCM research (Chen S. et al., 2016). Recently, a pharmacology-based systemsDock network was developed to predict and assess the bioactive sites between the components and single molecules in order to comprehensively explore the interactions among components and evaluate the pharmacological effects of natural plants (Bai and Abernethy, 2013). Therefore, we used this network to predict the potential binders of *R. uniflorum* in OSCC to investigate the effects of the *R*. *uniflorum* ethyl acetate (RUEA) extracts on cell proliferation, apoptosis, invasion, migration and the EMT process *in vitro* and *in vivo.*

## Materials and Methods

### Materials and Sample Preparation

The radix of *R. uniflorum* (L.) DC. samples were collected in Henan Province, China. The original specimen was deposited for future use, and the RUEA extracts were dissolved in dimethyl sulfoxide (DMSO) and diluted to obtain two different storage concentrations (50 μg/mL and 50 mg/mL). The extracts were stored at 4°C and diluted to the required concentration before use.

### Ultra-Performance Liquid Chromatography-Q/Time-of-Flight Mass Spectrometry (UPLC-Q/TOF-MS) Analysis

Chromatographic analysis was performed using an Acquity UPLC system (Waters, Milford, MA, United States) with a 2 μL injection volume. Next, Tandem MS was performed using a Q-TOF mass spectrometer (Waters) with an electrospray ionization interface. The results were analyzed using MassLynx v4.1 software (Waters).

### SystemsDock and Prediction of Related Proteins

Pharmacology-based network prediction and analysis were performed using systemsDock^1^. The required processes were divided into three main steps: (i) Selection of ROS-dependent signaling proteins (in SBML format) in OSCC from literature surveys (Tang, 2009, unpublished data); (ii) Uploading structure files based on the results of UPLC-Q/TOF-MS analysis in PubChem; (iii) Obtaining the prediction results and screening out the most interesting proteins.

### Cell Culture

Human OSCC cell line SCC15 (American Type Culture Collection) was cultured in Dulbecco’s modified Eagle medium/Nutrient Mixture F-12 medium (Gibco, United States) containing 12.5% fetal bovine serum (Gibco) and cultured at 37°C incubator containing 5% CO_2_.

### Plasmid Construction and Cell Transfection

SCC15 cells were transfected with shRNA Prx1 plasmid (Santa Cruz Biotechnology, Santa Cruz, CA, United States) using Lipofectamine 2000 (Invitrogen, Life Technologies Corp., Carlsbad, CA, United States). The plasmid was constructed according to standard techniques, and the target sequence for the Prx1 shRNA was: 5′-CGAAGCGCACCAATTGCTCA-3′. The shRNA Plasmid-A (Santa Cruz Biotechnology) was used as a vector control. The efficiency of Prx1 knockdown was determined by reverse transcription polymerase chain reaction (RT-PCR) and western blotting after selection with puromycin.

### Cell Proliferation Assay

SCC15 cells were treated with RUEA extracts at the concentrations of 0 (vehicle control), 12.5, 25, 50, and 100 μg/mL for 24, 48, and 72 h. Cell viability was detected by using Cell Counting kit-8 (CCK-8; Dojindo, Tokyo, Japan). Cell viability (%) was presented as [OD] test wells/[OD] control wells × 100%.

### Wound Healing Assay

SCC15 cells treated with 0, 12.5, 25, and 50 μg/mL RUEA extracts for 24 and 48 h were seeded into 6-well plates (5 × 10^5^ cells/well). Scratch wounds were made through the cell monolayer with a 200 μL pipette tip in each well. Wound distance was measured in three locations for each well at 0, 24, and 48 h.

### Matrigel Invasion Assay

Cell invasion assay was performed using 24-well Transwells (8 μm pore size; Corning, NY, United States) coated with Matrigel (100 μL per well, thickness: 3 mm; Becton Dickinson, San Jose, CA, United States) in triplicate. SCC15 cells were treated with 0, 12.5, 25, and 50 μg/mL RUEA for 24 and 48 h. Cells (10^5^ well) were resuspended in serum-free medium, plated into the upper chamber, and then incubated for 24 h with complete culture medium added to the lower chamber. Following incubation, cells inside the chamber were wiped off with a cotton swab. The invading cells which stuck to the lower side of the filter membrane were stained with Hematoxylin (Salarbio, Beijing, China) and examined using a microscope (Olympus, Tokyo, Japan).

### Apoptosis Analysis

SCC15 cells were treated with 0, 12.5, 25, 50, and 100 μg/mL RUEA extracts for 24 and 48 h. Cells were then resuspended and incubated with 5 μL of Annexin V-fluorescein isothiocyanate (FITC) for 15 min, and then incubated with 5 μL propidium iodide (PI) for 5 min in the dark. Apoptosis rates were analyzed by flow cytometry (FACSVerse, BD, San Jose, CA, United States).

### Quantitative Real-Time PCR

Total RNA was extracted from SCC15 cells using TRIzol (Invitrogen). cDNA was synthesized using a HiFi-MMLV cDNA Kit (Cwbiotech, Beijing, China). Real-time PCR reaction was performed using UltraSYBR Mixture (Cwbiotech) according to the manufacturer’s protocol. Primers were as follows: Prx1, 5′-GGGTATTCTTCGGCAGATCA-3′ and 5′-TCCCCATGTTTGTCAGTGAA-3′; E-cadherin, 5′-TTGCTACTGGAACAGGGACA-3′ and 5′-GTATTGGGAGGAAGGTCTGC-3′; vimentin, 5′-GAAGAGAACTTTGCCGTTGA-3′ and 5′-CGAAGGTGACGAGCCATT-3′; Snail, 5′-TTACCTTCCAGCAGCCCTAC-3′ and 5′-GACAGAGTCCCAGATGAGCA-3′, and GAPDH, 5′-AGGTCGGTGTGAACGGATTTG-3′ and 5′-TGTAGACCATGTAGTTGAGGTCA-3′. PCR was performed in triplicate, and fold enrichment was calculated with the – ΔΔCt method relative to the expression of GAPDH.

### Western Blotting

Proteins from SCC15 cells and xenograft tumor tissues were extracted using immunoprecipitation assay buffer. The concentration of total protein was determined using the Lowry method. Equal amounts of protein were separated by sodium dodecyl sulfate polyacrylamide gel electrophoresis on 12% gels and transferred to nitrocellulose membranes. The membranes were then blocked and incubated with primary antibodies against Prx1 (1:1000; Abcam, Cambridge, MA, United States), Snail (1:500; Abcam), E-cadherin (1:1000; Cell Signaling Technology, Beverly, MA, United States), vimentin (Bioss, Beijing, China), and GAPDH (1:2000; Sigma–Aldrich, United States). The protein bands were detected with horseradish peroxidase (HRP)-conjugated secondary antibodies and visualized using an enhanced chemiluminescence detection system.

### Tumor Xenograft Mouse Model

All procedures performed in studies involving animals were carried out in accordance with the ethical standards of the Ethics Committee of Capital Medical University School of Stomatology (Approval No. KQYY-201604-010). Female BALB/c-NU mice (4–5 weeks old, 16–20 g) were purchased from Beijing Vital River Laboratory Animal Technology Co. (Beijing, China). SCC15 cells (5 × 10^6^/100 μL/mouse) were subcutaneously injected into the backs of mice, and xenografts were successfully formed after 1 week. All mice with xenografts were randomly separated into four groups: blank control group, vehicle control group, 25 mg/kg RUEA group and 250 mg/kg RUEA group. Mice in the blank control group were untreated, whereas mice in other groups were treated with 100% DMSO (5 mL/kg), 25 mg/kg RUEA extracts and 250 mg/kg RUEA extracts, respectively. Treatments were given via intratumoral injection every 4 days in the morning. During this period, tumor length, tumor width and body weight were recorded every 4 days. The tumor volume (TV) was calculated using the formula: TV = π/6 × length × (width)^2^. At the end of the study (5 weeks after inoculation), all mice were sacrificed by cervical dislocation. Then, all tumors were removed and each single tumor was cut in two parts; one part was fixed with 10% neutral formalin for immunohistochemical staining, and the other was stored in liquid nitrogen for western blot analysis.

### Immunohistochemistry

The tumors were collected, fixed with 10% neutral formalin for 24 h, embedded in paraffin, serially sectioned at 4 μm and processed for immunohistochemical staining. After antigen retrieval conducted with citrate buffer (pH = 6.0), the sections were treated with protein block solution (Fuzhou Maixin Biotech, China) for 15 min at 37°C and incubated with anti-Ki67 antibody (Fuzhou Maixin Biotech, China) overnight at 4°C. Positive cells were detected with HRP-conjugated secondary antibody and visualized using 3,3′-diaminobenzidine (DAB) staining. Hematoxylin was used as the counterstain. The proliferation rate of each tumor was evaluated by estimating the average percentage of Ki67 positive cells among total epithelial cells in five random areas of a section under 200× magnification.

### Terminal Deoxynucleotidyl Transferase dUTP Nick-End Labeling (TUNEL) Assay

The apoptotic cells in tumor sections were detected using TUNEL assay. Sections were dewaxed, hydrated, and incubated with proteinase K at 37°C for 15 min, and washed with phosphate-buffered saline three times. Sections were then incubated with 50 μL of TUNEL reaction mixture and 50 μL of converter-POD at 37°C for 60 and 30 min, respectively. Freshly prepared DAB solution was incubated with sections for 20 min. The apoptotic cells were photographed and counted by Image Pro.

### Statistical Analysis

Experiments were conducted in triplicates. Differences with *P*-values of less than 0.05, analyzed by one-way analysis of variance (SPSS v17.0), were considered statistically significant.

## Results

### Identification of the Chemical Composition of RUEA Extracts by UPLC-Q/TOF-MS

**FIGURE 1:**
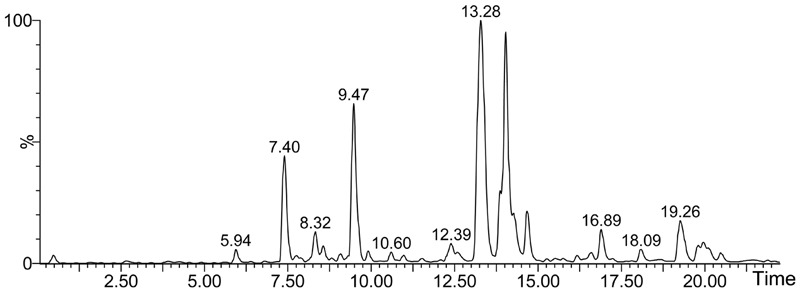
Base peak ion (BPI) chromatogram of extracts from *Rhaponticum uniflorum* roots in negative ion mode determined by ultra-performance liquid chromatography-Q/-time-of-flight-mass spectrometry (UPLC-Q/TOF-MS).

**Figure 1** shows the base peak ion (BPI) chromatogram of RUEA extracts. A total of 14 compounds were tentatively identified based on retention time, molecular ions, major fragment ions, and previously published articles and online databases. The identified compounds were mainly classified as phytoecdysteroids and triterpenoids. The details of the identified compounds are listed in **Table 1**.

**Table 1:** Chemical composition of roots from *Rhaponticum uniflorum* determined by UPLC-Q/TOF-MS.

No.	*t*_R_ (min)	Molecular	Identification	[M–H]^-^	[M–H]^-^	[M+HCOO]^-^
		formula		theoretical	experimental	
1	5.94	C_27_H_44_O_8_	Turkesterone	495.2958	495.2957	541.3032
2	6.75	C_19_H_26_O_5_	Rubrosterone	333.1702	333.1811	379.1746
3	7.38	C_27_H_44_O_8_	Rhapontisterone	495.2958	495.2988	
4	7.39	C_27_H_44_O_7_	Epibrassinolide	479.3009	479.3031	525.3065
5	7.75	C_27_H_44_O_7_	Ajugasterone C	479.3009	479.3004	525.3085
6	8.55	C_29_H_48_O_8_	Rhapontisterone C	523.3271	523.2935	
7	9.07	C_29_H_46_O_8_	Viticosteron E	521.3114	521.3143	567.3157
8	9.46	C_27_H_44_O_7_	Ecdysterone	479.3009	479.3056	525.3094
9	9.97	C_29_H_48_O_7_	Rhapisterone	507.3322	507.3334	567.3153
10	10.97	C_27_H_42_O_6_	Stachysterone B or its isomer	461.2903	461.2947	507.2996
11	13.32/14.03/14.67		Unknown		329.2489	
12	16.13	C_35_H_56_O_8_	Ziyu glycoside	603.3897	603.3752	
13	16.89	C_36_H_58_O_9_	28-Glucosylpomolate	633.4003	633.3902	
14	18.07	C_33_H_54_O_13_	Ecdysterone glucopyranoside	641.3537	641.4529	
15	19.26	C_33_H_54_O_13_	Ecdysterone glucopyranoside isomer	641.3537	641.4491	

*t_*R*_, retention time.*

### Pharmacology-Based Network Prediction of RUEA Extracts Binding Proteins

**FIGURE 2:**
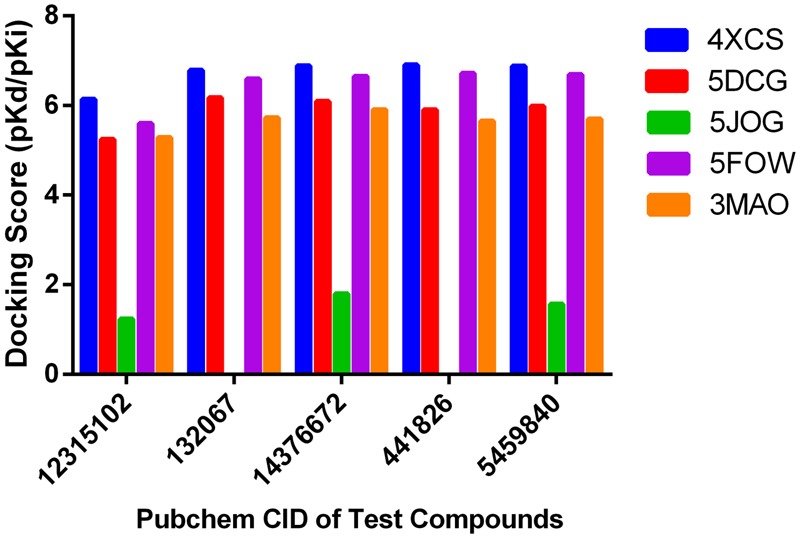
Docking scores of pharmacology-based network predictions. Docking scores for the test compounds (14376672: Turkesterone, 12315102: Rubrosterone, 132067: Rhapontisterone, 441826: Ajugasterone C, 5459840: Ecdysterone; 4XCS: peroxiredoxin1, 5DCG: glutathione *S*-transferase Pi, 5J0G: SOD1, 5FOW: ATOX1, 3MAO: selenoprotein X1).

SystemsDock is a web server for network pharmacology-based prediction and analysis. It has an elaborately designed scoring function for molecular docking to evaluate protein–ligand binding potential. **Figure 2** shows docking scores and predicted binding affinities for target proteins. Compounds of RUEA extracts searched on SystemsDock are listed in **Table 2**. Among the interesting ROS-related proteins, Prx1 (PDB: 4XCS; score avg.: 7.07) was identified as a potential binder.

**Table 2:** Two-dimensional structures of chemical constituents from the roots of *Rhaponticum uniflorum* for pharmacology-based prediction and analysis.

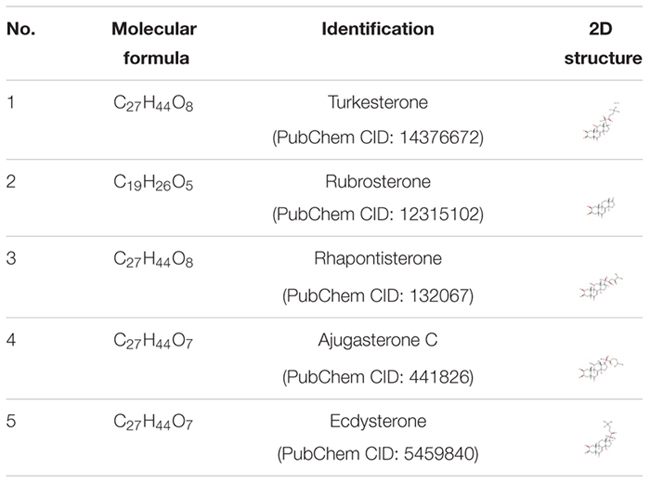

### RUEA Extracts Inhibit Proliferation and Induce Apoptosis in SCC15 Cells

**FIGURE 3:**
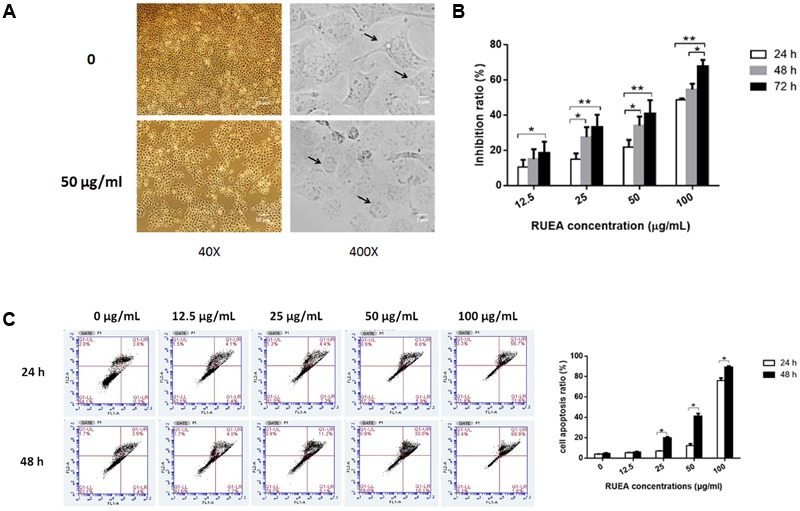
*Rhaponticum uniflorum* ethyl acetate (RUEA) extracts inhibited cell proliferation and induced apoptosis in SCC15 cells. **(A)** Morphological changes in SCC15 cells. **(B)** CCK8 assay of the effect of RUEA extracts on SCC15 cells for 24, 48, and 72 h at concentrations of 0 (vehicle control), 12.5, 25, 50, and 100 μg/mL. **(C)** Annexin V-FITC/PI double-staining for detection of apoptosis following treatment with RUEA extracts (0, 12.5, 25, 50, and 100 μg/mL) for 24, 48, and 72 h in SCC15 cells. Data representing three independent experiments are shown as means ± SDs. ^∗^*P* < 0.05; ^∗∗^*P* < 0.01.

Compared with the control group, SCC15 cells treated with 50 μg/mL RUEA extracts grew slowly and turned from polygonal to round (**Figure 3A**). To determine the effects of RUEA extracts on cell proliferation and apoptosis in SCC15 cells, cells were treated with 0, 12.5, 25, 50, and 100 μg/mL of RUEA extracts for 24, 48, and 72 h. Cell viability was measured by CCK8 assay and cell apoptosis rates were analyzed by flow cytometry. As shown in **Figure 3B**, cell viability was significantly decreased after RUEA extract treatment in a concentration-dependent manner. Annexin V-FITC/PI double-staining was used for the detection of different phases of apoptotic cells. The results showed that the proportions of the early and terminal phase of apoptotic cells increased after RUEA extract treatment in a concentration-dependent manner at 24 and 48 h, respectively (**Figure 3C**).

### RUEA Extracts Suppress Migration and Invasion in SCC15 Cells

**FIGURE 4:**
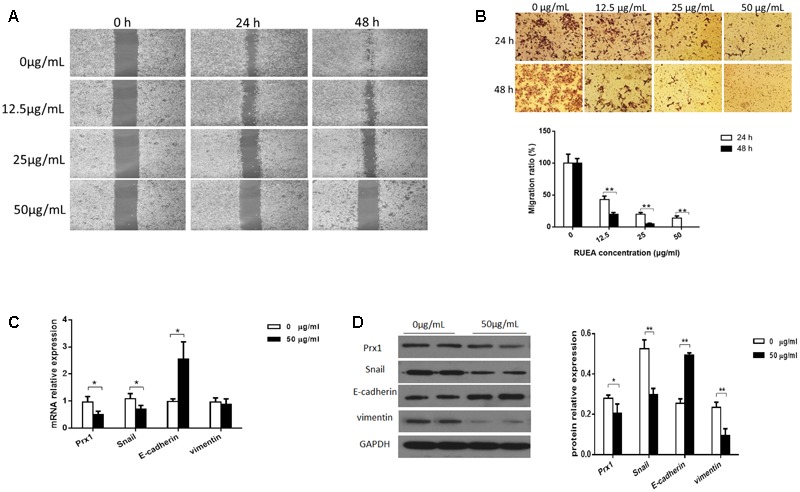
*Rhaponticum uniflorum* ethyl acetate extracts suppressed invasion and migration in SCC15 cells and regulated the epithelial-to-mesenchymal transition (EMT). **(A)** Wound healing assay to examine the effects of RUEA extracts on SCC15 cell mobility. **(B)** Images of SCC15 cells that penetrated through filters to the other side of inserts (upper panel) and statistical analysis (lower panel). **(C)** Expression of Prx1, E-cadherin, vimentin, and Snail mRNAs in RUEA extract-treated SCC15 cells. **(D)** Representative western blots from one of three separate experiments for expression of Prx1, E-cadherin, vimentin, and Snail proteins in RUEA extract-treated SCC15 cells. Data are presented as means ± SDs. ^∗^*P* < 0.05; ^∗∗^*P* < 0.01.

SCC15 cells were treated with 0, 12.5, 25, and 50 μg/mL RUEA extracts for 24 and 48 h, wound healing and Matrigel invasion assays were performed. Wound healing assays showed that the open wound area decreased significantly in RUEA extract treated cells in a concentration-dependent manner compared with that in control cells (**Figure 4A**). Similarly, RUEA extracts significantly decreased the number of invasive cells after 24 and 48 h (**Figure 4B**).

### RUEA Extracts Suppress Prx1 and the EMT Process in SCC15 Cells

The EMT process accelerates cancer metastasis by increasing cell migration and invasion. To determine whether the EMT process was modulated by RUEA extracts, we evaluted the expression of E-cadherin, vimentin, and Snail in SCC15 cells treated with 50 μg/mL RUEA extracts for 48 h. We also evaluated the expression of Prx1, which was identified as a potential binder of RUEA extracts. Our data showed that at the mRNA level, RUEA extracts significantly increased the expression of E-cadherin and decreased the expression of Prx1 and Snail compared with the control group (**Figure 4C**). At the protein level, RUEA extracts significantly increased the expression of E-cadherin and decreased the expression of Prx1, vimentin, and Snail compared with the control group (**Figure 4D**).

To further explore whether RUEA extracts suppressed cell migration and invasion via Prx1 in oral cancer, Prx1 knockdown SCC15 cells were established. RUEA extracts suppressed cell invasion and migration in Prx1 knockdown SCC15 cells treated with 0, 12.5, 25, and 50 μg/mL RUEA extracts for 24 and 48 h. Moreover, RUEA extracts inhibited the expression of E-cadherin, vimentin, and Snail in Prx1 knockdown SCC15 cells for 48 h (**Supplementary Figure S1**), indicating that RUEA extracts may target other molecules in addition to Prx1 to inhibit cell migration and invasion in oral cancer.

### RUEA Extracts Inhibit Tumor Growth in an OSCC Xenograft Model

**FIGURE 5:**
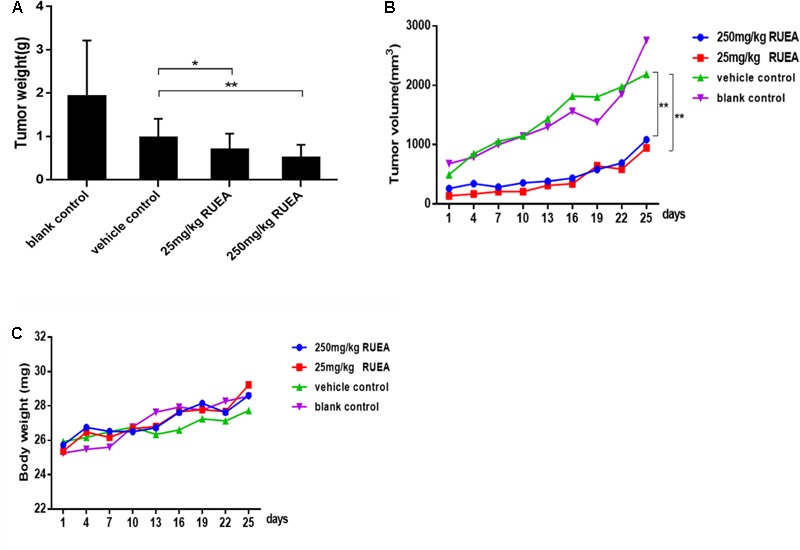
*Rhaponticum uniflorum* ethyl acetate extracts inhibited tumor growth in an oral squamous cell carcinoma (OSCC) xenograft model. **(A)** Average tumor weight. **(B)** Tumor growth curves, and **(C)** body weight curves in mice treated with RUEA extracts (25 and 250 mg/kg), vehicle control, and blank control groups. Data are presented as means ± SDs (*n* = 6). ^∗^*P* < 0.05; ^∗∗^*P* < 0.01.

To further confirm the anti-tumor effects of RUEA extracts *in vivo*, we established an OSCC xenograft model. Compared with the control group, mice in 25 and 250 mg/kg of RUEA extracts groups showed significantly decreased tumor weights (**Figure 5A**) and TVs (**Figure 5B**). However, there were no significant difference in tumor weights or TVs between the 25 and 250 mg/kg groups. Notably, the body weights of all mice remained stable, and there were no significant differences among groups (**Figure 5C**).

### RUEA Extracts Suppress Cell Proliferation and Induces Apoptosis *in Vivo*

**FIGURE 6:**
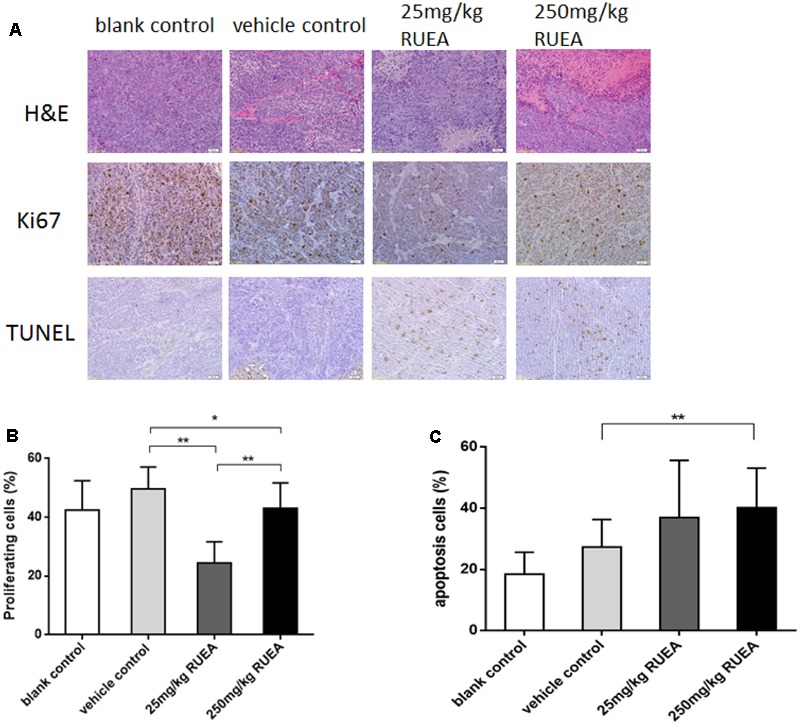
*Rhaponticum uniflorum* ethyl acetate extracts decreased cell proliferation and induced apoptosis in an OSCC xenograft model. **(A)** Hematoxylin and eosin (H&E) staining, immunohistochemical analysis of Ki67 and TUNEL staining of apoptotic cells in OSCC xenograft tissues. **(B)** Cell proliferation rates. **(C)** Apoptosis rates in xenograft tissues of the different groups. Data are presented as means ± SDs. ^∗^*P* < 0.05; ^∗∗^*P* < 0.01.

Hematoxylin and eosin staining revealed that necrotic tissues, abnormal mitoses, and shrinking nuclei were increased after RUEA extract treatment (**Figure 6A**).

As shown in **Figures 6A,B**, RUEA extract treatment significantly suppressed cell proliferation. The positive rates of Ki-67 were 49.6, 22.3, and 43.2% in the vehicle control group, 25 and 250 mg/kg RUEA groups, respectively. Moreover, the proliferation rate was higher in the 250 mg/kg group than in the 25 mg/kg group. No differences were found between the vehicle control group and the blank control group.

Next, we performed TUNEL assay to observe the apoptotic cells in the tumors. As shown in **Figures 6A,C**, RUEA extract treatment induced cell apoptosis. The average cell apoptosis rate in the vehicle control group was 27.4% which was much higher than in the blank control group. Moreover, apoptosis rates reached as high as 36.9 and 40.2% in the 25 and 250 mg/kg groups, respectively. Thus, our results suggested that RUEA reduced cell proliferation and induced apoptosis in OSCC.

### RUEA Extracts Inhibit Prx1 Expression and the EMT Process *in Vivo*

**FIGURE 7:**
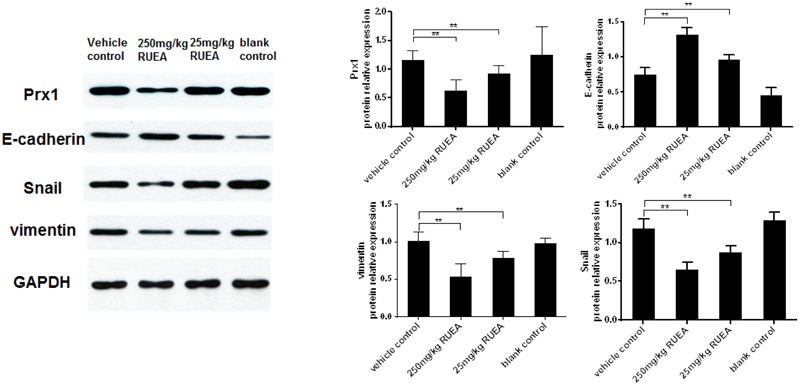
*Rhaponticum uniflorum* ethyl acetate extracts inhibited the EMT in an OSCC xenograft model. Representative western blots for Prx1, E-cadherin, vimentin, and Snail expression in xenograft tissues of different groups are shown. Data are presented as means ± SDs. ^∗^*P* < 0.05; ^∗∗^*P* < 0.01.

Western blot results showed that compared with the vehicle control group, RUEA extract treatment obviously decreased Prx1 expression in the transplanted tumors. As shown in **Figure 7**, compared with the vehicle control group, RUEA extracts increased E-cadherin expression and decreased vimentin and Snail expression in the transplanted tumors. No significant differences were observed between the two control groups. Our results suggested RUEA extracts inhibited Prx1 expression and the EMT process *in vivo*.

## Discussion

Surgery, radiotherapy, and chemotherapy are major treatment options for patients with OSCC. However, the effects of traditional therapies for OSCC are not satisfactory. The researchers have been attempting to develop novel, efficient anti-cancer agents to meet this urgent need. Many studies have shown that some herbal products have obvious effects on the inhibition of tumor growth and metastasis in various cancers (Wang et al., 2010). *R. uniflorum* has been used as an herbal medicine to treat inflammation and tumors and improve immunological functions in China (Zhang et al., 2005; Li et al., 2009). Moreover, Jin et al. (2011) found that RUWEs could inhibit the growth of H22 hepatocarcinoma cells. A recent study showed that *Rhapontici radix* ethanol extract has an anti-inflammatory effect by activating nuclear factor-κB and mitogen activated protein kinase signaling pathways (Jeong et al., 2016). In our previous study, we found that SCC15 cells grew slowly and that their cell morphology changed from a polygonal shape to a round shape after treatment with 50 μg/mL RUEA extracts for 48 h (Chen H. et al., 2016). In the present study, we expanded these findings and showed that RUEA extracts reduced cell proliferation, induced apoptosis, and modulated tumor growth in an xenograft mouse model. Thus, our findings provided important insights into the effects of this TCM on OSCC progression. In this study, Ki67 scores decreased and the apoptosis rates increased after RUEA extract treatment for 25 days. Notably, the tumor cells proliferated more rapidly in the 250 mg/kg group, suggesting that there may be more necrotic tissues in the tumors in this group. Interestingly, inflammation can induce Ki-67 overexpression in some diseases (Hafez and Tahoun, 2011). Oxidative stress can induce DNA injury and regulate the progression of various cancers (Zhang et al., 2017). Indeed, Saed et al. (2017) found that oxidative stress was involved in carcinogenesis and metastasis by inducing phenotypic modifications in ovarian cancer cells. Our previous study also showed that OSCC cells elevated intracellular ROS level compared with those in oral precancerous lesion cells. Importantly, the roots of *R. uniflorum* have antioxidant activity (Jin et al., 2011; Jeong et al., 2016). Yan et al. (2013) showed that RUWEs enhanced the total antioxidant capacity, increased scavenging activity of free hydroxyl radicals, and regulated superoxide dismutase activity. These findings suggest that RUEA extracts may inhibit tumor growth and induce apoptosis by modulating oxidative stress in OSCC.

Invasion and metastasis are primary causes of mortality in many cancers including OSCC. Studies have suggested that the EMT process is a critical step for tumor invasion and metastasis and may become an indicator of progression and prognosis indicator as well as a therapeutic target in some cancers (Chaw et al., 2012). During the EMT process, epithelial cells acquire the features of mesenchymal cells by losing intracellular adhesion structure, and polarity, and subsequently gaining invasive ability (Hay, 2005). In the current study, RUEA extracts suppressed SCC15 cell invasion and migration after treatment for 24 and 48 h. The expression of E-cadherin was upregulated, whereas the expression of vimentin and Snail was downregulated in SCC15 cells treated with RUEA extracts. Similarly, RUEA extracts influenced the EMT *in vivo*, which resulted in elevated E-cadherin expression and decreased vimentin and Snail expression in the transplanted tumors, indicating that RUEA extracts inhibit cell migration and invasion by hindering the EMT process in OSCC.

Network pharmacology, the theoretical analysis of biological network performance, provides a new method to explore the multi-targeted effects of drugs in TCM research (Bai and Abernethy, 2013). Molecular virtual docking can be used to rapidly calculate and predict the binding potential of a small molecule via the computational methods (Hsin et al., 2016). In this study, we used UPLC-Q/TOF-MS analysis and detected 14 constituents, including ecdysterone, rhapontisterone, turkesterone, and ziyu-glycosides. Feng et al. (2014) found that ecdysterone was one of the main components of RUEA extracts, had estrogen-like activity, and played an important role in oxidative damage induced by H_2_O_2_ in B3 human lens epithelial cells. In this study, application of molecular virtual docking to predict potential binding partners or known targets from some oxidative stress-related proteins showed that Prx1 was a potential binding protein combined with ecdysterone in RUEA extracts. Prx1 is a peroxide-detoxifying enzyme that scavenges ROS. The molecular chaperone activities of Prx1 are enhanced under oxidative stress conditions, and Prx1 regulates the intracellular balance of cell survival and apoptosis to modulate cell proliferation (Neumann et al., 2009). The expression of Prx1 is increased in various malignant tumors, including OSCC, and related to patient prognosis. Accordingly, our previous studies showed that Prx1 and oxidative injury may be involved in the pathogenesis of oral leukoplakia and OSCC (Zhang et al., 2015). The invasion and migration of SCC15 cells are increased by Prx1 overexpression and reduced by Prx1 silencing (Niu et al., 2016). Moreover, Prx1 modulates the EMT process in tobacco-related OSCC (Zhang et al., 2014). In the present study, compared with the control group, RUEA extracts downregulated the expression of Prx1 in SCC15 cells and transplanted tumors. RUEA extracts also inhibited the EMT program, migration, and invasion in SCC15 cells, even in the absence of Prx1. These results suggest that RUEA extracts suppress tumor growth and invasion by inhibiting Prx1 and EMT progress in OSCC. RUEA extracts may have more complicated mechanism in hindering tumor growth and invasion in oral cancer. Further studies are needed to fully elucidate the mechanisms involved in this process.

## Conclusion

Our study identifies, for the first time, that RUEA extracts effectively inhibit tumor growth and invasion in OSCC by suppressing Prx1 expression and the EMT process in OSCC. Our findings provide a basis for further studies of RUEA extracts in the treatment of oral cancer.

## Author Contributions

XT contributed to the conception and analysis of the study and revision of manuscript. LH contributed to the conception and analysis of the study. HC performed most of experiments and contributed to data analysis and manuscript writing. CW and MQ performed experiments *in vitro*. ZT and LG contributed to establishment of the animal model and pretreated tissues. JL contributed to the analysis of RUEA extract components. MW and MZ helped perform the analysis with constructive discussion. All the authors are in agreement with the content of the manuscript.

## Conflict of Interest Statement

The authors declare that the research was conducted in the absence of any commercial or financial relationships that could be construed as a potential conflict of interest.

## Footnotes

1 http://systemsdock.unit.oist.jp/

## Supplementary Material

FIGURE S1 *Rhaponticum uniflorum* ethyl acetate (RUEA) extracts regulated cell invasion and migration through modulating the EMT Process in Prx1 knockdown (shPrx1) SCC15 cells. **(A)** mRNA and protein expression of Prx1 in vector control and shPrx1 SCC15 cells. **(B)** Wound healing assay to examine the effects of RUEA extracts on cells mobility. **(C)** Images of RUEA extract-treated vector control and shPrx1 SCC15 cells that penetrated through filters to the other side of inserts (Upper) and statistical analysis (Lower). **(D)** Representative western blots from one of three separate experiments for protein expression of E-cadherin, vimentin, and Snail in RUEA extract-treated vector control and shPrx1 SCC15 cells. Data are presented as means ± SDs. ^∗^*P* < 0.05; ^∗∗^*P* < 0.01.

## References

[B1] Bai J. P. Abernethy D. R. (2013). Systems pharmacology to predict drug toxicity: integration across levels of biological organization. *Annu. Rev. Pharmacol. Toxicol.* 53 451–473. 10.1146/annurev-pharmtox-011112-140248 23140241

[B2] Cha M. K. Suh K. H. Kim I. H. (2009). Overexpression of peroxiredoxin I and thioredoxin1 in human breast carcinoma. *J. Exp. Clin. Cancer Res.* 28:93. 10.1186/1756-9966-28-93 19566940 PMC2711968

[B3] Chaw S. Y. Majeed A. A. Dalley A. J. Chan A. Stein S. Farah C. S. (2012). Epithelial to mesenchymal transition (EMT) biomarkers–E-cadherin, beta-catenin, APC and Vimentin–in oral squamous cell carcinogenesis and transformation. *Oral Oncol.* 48 997–1006. 10.1016/j.oraloncology.2012.05.011 22704062

[B4] Chen H. Wang C. X. Zhang M. Tang X. F. (2016). Effect of Radix rhapontici on the expression of transcription factor Ets-1 and Prx1 in oral cancer. *Beijing J. Stomatol.* 24 83–86.

[B5] Chen S. Jiang H. Cao Y. Wang Y. Hu Z. Zhu Z. (2016). Drug target identification using network analysis: taking active components in *Sini* decoction as an example. *Sci. Rep.* 6:24245. 10.1038/srep24245 27095146 PMC4837341

[B6] Chow M. S. Huang Y. (2010). Utilizing Chinese medicines to improve cancer therapy–fiction or reality? *Curr. Drug Discov. Technol*. 7:1 10.2174/157016310791162721 20230363

[B7] Feng C. Y. Huang X. R. Qi M. X. Tang S. W. Chen S. Hu Y. H. (2014). Mitochondrial proteomic analysis of ecdysterone protection against oxidative damage in human lens epithelial cells. *Int. J. Ophthalmol.* 7 38–43. 10.3980/j.issn.2222-3959 24634861 PMC3949456

[B8] Hafez N. H. Tahoun N. S. (2011). Diagnostic value of p53 and Ki-67 immunostaining for distinguishing benign from malignant serous effusions. *J. Egypt. Natl. Canc. Inst.* 23 155–162. 10.1016/j.jnci.2011.11.001 22776843

[B9] Hay E. D. (2005). The mesenchymal cell, its role in the embryo, and the remarkable signaling mechanisms that create it. *Dev. Dyn.* 233 706–720. 10.1002/dvdy.20345 15937929

[B10] Hsin K. Y. Matsuoka Y. Asai Y. Kamiyoshi K. Watanabe T. Kawaoka Y. (2016). SystemsDock: a web server for network pharmacology-based prediction and analysis. *Nucleic Acids Res.* 44 W507–W513. 10.1093/nar/gkw335 27131384 PMC4987901

[B11] Jeong Y. H. Oh Y. C. Cho W. K. Yim N. H. Ma J. Y. (2016). Anti-inflammatory effect of Rhapontici radix ethanol extract via inhibition of NF-κB and MAPK and induction of HO-1 in macrophages. *Mediators Inflamm.* 2016:7216912. 10.1155/2016/7216912 27524868 PMC4976174

[B12] Jin A. H. Xu H. X. Liu W. J. Quan J. S. Zhe X. Y. (2011). Studies on anti-tumor effect and mechanism of *Rhaponticum uniflorum* in H22-bearing mice. *Chin. J. Exp. Tradit. Med. Form.* 5 165–167.

[B13] Kim J. H. Bogner P. N. Baek S. H. Ramnath N. Liang P. Kim H. R. (2008). Up-regulation of peroxiredoxin 1 in lung cancer and its implication as a prognostic and therapeutic target. *Clin. Cancer Res.* 14 2326–2333. 10.1158/1078-0432 18413821

[B14] Li Y. T. Li L. Chen J. Hu T. C. Huang J. Guo Y. W. (2009). 7-Chloroarctinone-b as a new selective PPARgamma antagonist potently blocks adipocyte differentiation. *Acta Pharmacol. Sin.* 30 1351–1358. 10.1038/aps.2009.113 19684608 PMC4007185

[B15] National Ceremonial Committee (2005). *Pharmacopoeia of the People’s Republic of China* 8th Edn. Beijing: Chemistry Engineering Edition 257.

[B16] Neumann C. A. Cao J. Manevich Y. (2009). Peroxiredoxin 1 and its role in cell signaling. *Cell Cycle* 8 4072–4078. 10.4161/cc.8.24.10242 19923889 PMC7161701

[B17] Niu W. Zhang M. Chen H. Wang C. Shi N. Jing X. (2016). Peroxiredoxin 1 promotes invasion and migration by regulating epithelial-to-mesenchymal transition during oral carcinogenesis. *Oncotarget* 7 47042–47051. 10.18632/oncotarget.9705 27259998 PMC5216922

[B18] Saed G. M. Diamond M. P. Fletcher N. M. (2017). Updates of the role of oxidative stress in the pathogenesis of ovarian cancer. *Gynecol. Oncol.* 145 595–602. 10.1016/j.ygyno 28237618

[B19] Shu X. Mcculloch M. Xiao H. Broffman M. Gao J. (2005). Chinese herbal medicine and chemotherapy in the treatment of hepatocellular carcinoma: a meta-analysis of randomized controlled trials. *Integr. Cancer Ther.* 4 219–229. 10.1177/1534735405279927 16113029

[B20] Siegel R. Ma J. Zou Z. Jemal A. (2014). Cancer statistics. *CA Cancer J. Clin.* 64 9–29. 10.3322/caac.21208 24399786

[B21] Walk E. L. Weed S. A. (2011). Recently identified biomarkers that promote lymph node metastasis in head and neck squamous cell carcinoma. *Cancers* 3 747–772. 10.3390/cancers3010747 24212639 PMC3756388

[B22] Wang S. Penchala S. Prabhu S. Wang J. Huang Y. (2010). Molecular basis of traditional Chinese medicine in cancer chemoprevention. *Curr. Drug Discov. Technol.* 7 67–75. 10.2174/157016310791162794 20226002

[B23] Warnakulasuriya S. (2009). Global epidemiology of oral and oropharyngeal cancer. *Oral Oncol.* 45 309–316. 10.1016/j.oraloncology 18804401

[B24] Warnakulasuriya S. (2010). Living with oral cancer: epidemiology with particular reference to prevalence and life-style changes that influence survival. *Oral Oncol.* 46 407–410. 10.1016/j.oraloncology 20403722

[B25] Wu Y. Zhou B. P. (2009). *Epithelial–Mesenchymal Transition in Development and Diseases.* New York, NY: Springer 187–211.

[B26] Yan X. Zhao H. Guan Y. Song Y. Meng J. (2013). A study on the effect of ethanol extract of *Radix rhapontici* on erythrocyte immune function in rats. *Afr. J. Tradit. Complement. Altern. Med.* 10 538–541. 10.4314/ajtcam.v10i6.25 24311883 PMC3847398

[B27] Zhang L. Li L. Gao G. Wei G. Zheng Y. Wang C. (2017). Elevation of GPRC5A expression in colorectal cancer promotes tumor progression through VNN-1 induced oxidative stress. *Int. J. Cancer* 140 2734–2747. 10.1002/ijc.30698 28316092

[B28] Zhang M. Hou M. Ge L. Miao C. Zhang M. Jing X. (2014). Induction of peroxiredoxin 1 by hypoxia regulates heme oxygenase-1 via NF-κB in oral cancer. *PLOS ONE* 9:e105994. 10.1371/journal.pone.0105994 25162226 PMC4146557

[B29] Zhang M. Niu W. Zhang J. Ge L. Yang J. Sun Z. (2015). Peroxiredoxin 1 suppresses apoptosis via regulation of the apoptosis signal-regulating kinase 1 signaling pathway in human oral leukoplakia. *Oncol. Lett.* 10 1841–1847. 26622762 10.3892/ol.2015.3424 PMC4533595

[B30] Zhang Y. H. Zhang J. G. Xie J. M. Chen G. L. Cheng D. L. (2005). Triterpenes from root of *Rhaponticum uniflorum*. *Zhongguo Zhong Yao Za Zhi* 30 1833–1836. 16499021

[B31] Zhu L. Lu Y. Chen D. (1991). Composition of essential oil from inflorescences of *Rhaponticum uniflorum* (L.) DC. *Zhongguo Zhong Yao Za Zhi* 16 739–740. 1811670

