# Supplementary figures and images for: Anti-tumor Effect of Rhaponticum uniflorum Ethyl Acetate Extract by Regulation of Peroxiredoxin1 and Epithelial-to-Mesenchymal Transition in Oral Cancer
Source: Front Pharmacol. 2017 Nov 23;8:870. doi: 10.3389/fphar.2017.00870 (PMC5703707; doi:10.3389/fphar.2017.00870)

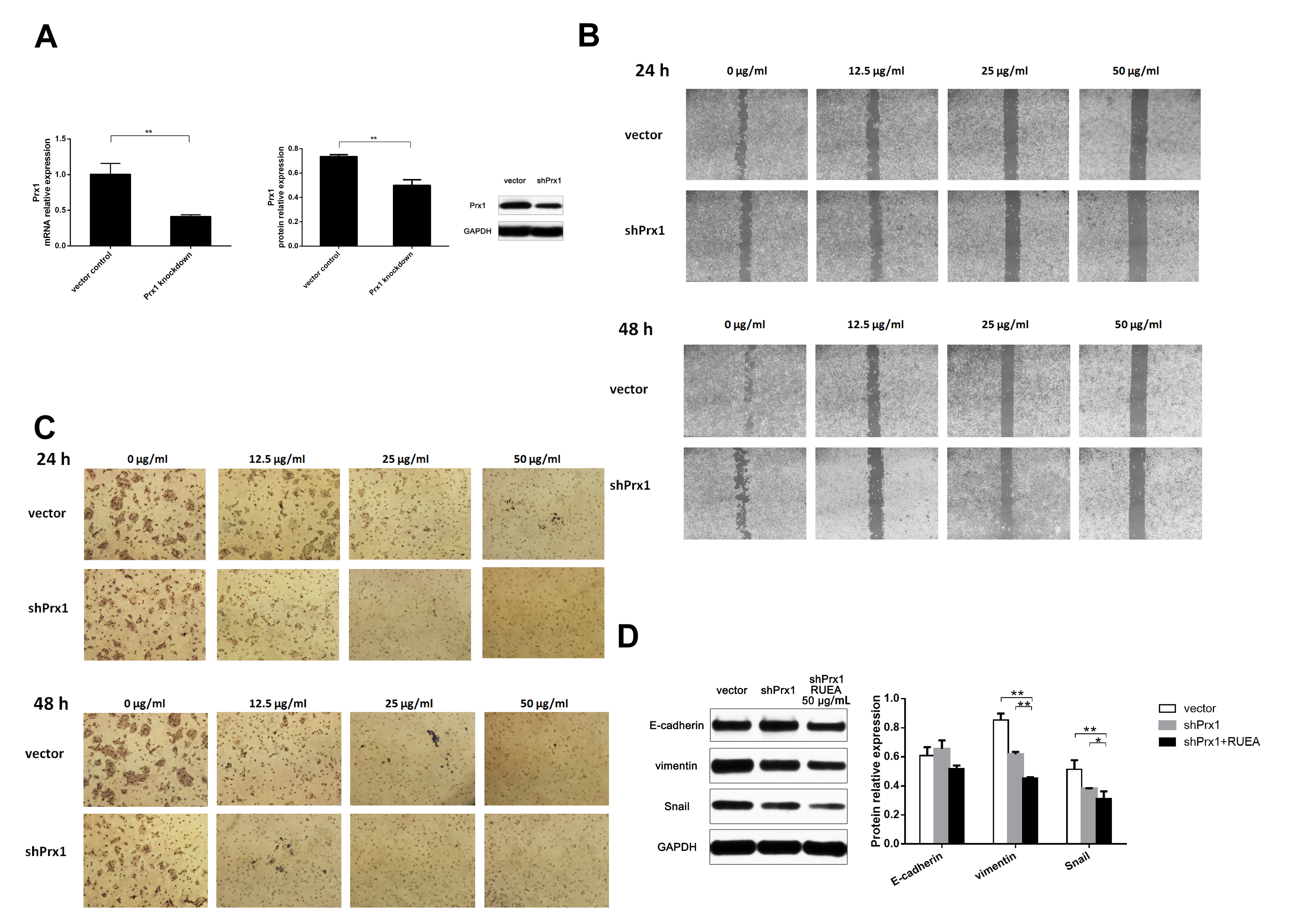

Supplement: FIGURE S1 — Rhaponticum uniflorum ethyl acetate (RUEA) extracts regulated cell invasion and migration through modulating the EMT Process in Prx1 knockdown (shPrx1) SCC15 cells. (A) mRNA and protein expression of Prx1 in vector control and shPrx1 SCC15 cells. (B) Wound healing assay to examine the effects of RUEA extracts on cells mobility. (C) Images of RUEA extract-treated vector control and shPrx1 SCC15 cells that penetrated through filters to the other side of inserts (Upper) and statistical analysis (Lower). (D) Representative western blots from one of three separate experiments for protein expression of E-cadherin, vimentin, and Snail in RUEA extract-treated vector control and shPrx1 SCC15 cells. Data are presented as means ± SDs. ∗P < 0.05; ∗∗P < 0.01. [file Image_1.JPEG]
